# A College for Specialties

**Published:** 1874-08

**Authors:** 


*MEDICAL AND SURGICAL REPORTER—July* 25, 1874:

A College for Specialties.—A few years ago some ambitious gentlemen in a northern city distributed a circular announcing the formation of a college for instruction in the specialties of medical science. The circular was, unfortunately, one might say, almost ludicrously worded, and was enough to quench the embryonic scheme at its very conception. Nevertheless, one who watches the rapid divarication of practice in all large cities, must acknowledge that specialism is bound to win in all such centers of competition, whatever the old heads say to the contrary.

It is the same in the old world; and the antagonism which the idea there encountered at one time, is giving way. The Paris correspondent of the *British Medical Journal* says, in a recent letter:

"Notwithstanding all that has been said and written on both sides of the channel in disparagement of specialism in medicine, specialists, in France, at least, are, whatever be the motives of the parties referred to, daily on the increase; and, as mentioned in one of my last letters, we have the approval of specialties by no less an authority than M. Depaul, Clinical Professor of Obstetrical Medicine and Surgery of the Faculty of Paris, who, in the introduction to the first number of the journal recently founded by him, sets himself up as an apologist for specialists, and declares that it is almost impossible for any man to be an 'encyclopædist.'"

This is eminently true, and there can be no question but that he who devotes himself to one limited territory of science can explore it more thoroughly than he who wanders over the whole realm of medicine.

The opposition to specialism arose largely from the notion that it meant ignorance of other branches, as great in proportion as it gave knowledge in the one. This is a mistake. The real specialist appreciates too well the numberless harmonies and secret sympathies of the economy to overlook or underrate the value of a broad and thorough knowledge of all its parts, functions and lesions. He will embrace all these in his ken, but bend this knowledge to one point, and upon one focus.

That a school will ultimately be demanded, where graduates from our colleges can have the opportunity to perfect themselves in one or the other branches, is obvious; and the more pertinent inquiry is, has that time yet arrived? Probably it has. Only a few of our largest cities can afford the requisite facilities and—abilities. There should be some place, short of Europe, where a physician can obtain a thorough course in ophthalmology, gynæcology, or any other special branch. As it is, he must enter the private office of some well known leader in the line he seeks, or he can get no opportunity in this country to acquire this training. Many practitioners are anxious to profit by such special studies; it is time an effort was made to teach them.

